# Measuring differences in the ERG in myopia using the RET*eval* device with skin electrodes

**DOI:** 10.1111/opo.13460

**Published:** 2025-02-13

**Authors:** Victoria Stapley, Roger S. Anderson, Kathryn Saunders, Pádraig J. Mulholland

**Affiliations:** ^1^ Centre for Optometry and Vision Science, Biomedical Sciences Research Institute Ulster University Coleraine UK; ^2^ National Institute for Health and Care Research (NIHR) Biomedical Research Centre at Moorfields Eye Hospital NHS Foundation Trust and UCL Institute of Ophthalmology, London, UK London UK

**Keywords:** electrophysiology, myopia, RET*eval*, visual function

## Abstract

**Introduction:**

Previous research suggests that the electroretinogram (ERG) is reduced and delayed in non‐pathological myopia. However, the invasive nature of the electrode and cumbersome equipment required has prevented the widescale uptake of ERG measures. This study investigated whether previously reported changes to the ERG response in myopia are also observable when measured using non‐invasive skin electrodes and a hand‐held ERG device.

**Method:**

Monocular flash ERGs were measured using the RET*eval*® device according to the ‘ISCEV 6 Step Dark First cd’ protocol in 46 participants with non‐pathological myopia (spherical equivalent refraction [SER] −0.50 to −11.25 D, median −3.75 D, median axial length [AL] 25.4 mm) and 47 non‐myopic controls (SER +2.00 to −0.25 D, median +1.00 D, median AL 23.6 mm). Measures were performed under pupil mydriasis with Sensor Strip skin electrodes.

**Results:**

The median implicit time for all dark‐adapted (DA) components was longer among myopes. Following Holm‐Bonferroni correction, this difference reached statistical significance (*p* < 0.05) for the DA 3.0 A‐wave, DA 10.0 A‐wave and B‐waves, and DA Oscillatory potentials 1 and 2. There were no significant differences between median light‐adapted (LA) implicit times nor response amplitudes between refractive groups. For all DA components, there was a significant, positive correlation between AL and implicit time (all *p* < 0.05).

**Conclusions:**

The RET*eval*®, used with skin electrodes, did not detect the reduction in ERG amplitude reported in myopic eyes using traditional ERG setups, potentially due to high inter‐subject variability and/or anatomical confounders associated with the use of a skin electrode. The RET*eval*® with skin electrodes did detect subtle delays to DA implicit times previously reported in myopia, with a positive relationship observed between AL and implicit time for all DA components. In contrast, no significant differences were observed for LA implicit times, which may indicate underlying differences in the dark‐adaptation process and/or scotopic visual pathways in myopia.

**Key points**

- Using the portable RET*eval®* and non‐invasive skin electrodes, a delay in dark‐adapted electroretinogram timing was found in myopia, with a positive relationship observed between axial length and implicit time.
- Delays in electroretinogram response timing for dark‐adapted but not light‐adapted conditions in myopia may indicate underlying differences in the dark‐adaptation process or scotopic visual pathway in myopia.
- No significant differences in response amplitude between myopes or non‐myopes, nor a statistically significant relationship between amplitude and axial length, were observed with the RET*eval®* and the use of non‐invasive skin electrodes.

## INTRODUCTION

Myopia is an ocular condition characterised by an eye which is too long for its optical power. Given the rising prevalence of myopia worldwide,^1^, ^2^, ^3^, ^4^, ^5^, ^6^ and the increased risk of sight‐threatening complications with increased myopia,^7^, ^8^, ^9^ it is important to enhance our understanding of how both the structure and function of the eye and visual pathways change in myopia, with a view to identifying biomarkers that may be used to detect, or even predict, progressive myopia. In particular, sensitive and precise endpoints are required to evaluate the efficacy and safety of novel myopic control interventions, such as low‐level red‐light therapy,^10^ ‘MyopiaX’ (by Dopavision, dopavision.com), and low‐dose atropine.^11^, ^12^

One area that has gained much interest is the objective assessment of retinal function using the electroretinogram (ERG). It records the electrical activity of retinal cells in response to different types of visual stimuli, such as full‐field flashes (ff‐ERG), pattern (*p*‐ERG), or multifocal (mf‐ERG) stimuli. In addition to the stimulus form, the resulting ERG waveform is dependent upon other stimulus parameters, such as luminance and frequency, as well as the adaptation status (photopic/scotopic) of the visual system. The International Society for Clinical Electrophysiology of Vision (ISCEV) provides standard ERG testing protocols to allow repeatable and comparable recordings across laboratories.

Non‐pathological myopia is widely acknowledged to lead to a reduction in the amplitude of the ff‐ERG response,^13^, ^14^, ^15^ with studies following ISCEV‐standard protocols reporting significant negative correlations between axial length and ERG amplitudes.^15^, ^16^, ^17^, ^18^ For example, Sachidanandam et al.^18^ found that with every 1 mm increase in axial length, there was a reduction of 15.7 and 23.4 μV in the dark‐adapted 3.0 ERG a‐wave and b‐wave amplitude, respectively. It should be noted that these relationships were derived from cross‐sectional data, rather than from a longitudinal data series tracking changes in axial length and ERG amplitude in individuals over time. Similar observations of reduced amplitude in myopia have also been found using *p*‐ERG^19^, ^20^ and mf‐ERG^16^, ^17^, ^18^, ^21^, ^22^, ^23^ and are thought to be a result of retinal changes in myopia, such as a reduction in neuron density^14^ or a subclinical reduction in their functional integrity.^13^

The impact of non‐pathological myopia on the timing of the ERG response is less clear. Some investigations have shown little or no difference between myopic and non‐myopic individuals when using the ff‐ERG,^13^, ^14^, ^15^, ^17^, ^18^ *p*‐ERG^19^ or mf‐ERG.^17^, ^22^ In contrast, other studies have observed delays in elements of the ff‐ERG,^16^ *p*‐ERG^20^, ^24^ and mf‐ERG^16^, ^21^, ^23^, ^25^, ^26^, ^27^, ^28^, ^29^ in individuals with myopia and/or with increasing axial length. Overall, the findings regarding the timing of the ERG response in relation to non‐pathological myopia are somewhat inconsistent, with some studies indicating no significant difference and others reporting delays in the response.

In terms of using ERG measures to predict myopia development, studies have observed that only children with a reduced mfERG response at baseline show more rapid progression.^30^, ^31^ Work by Jiang et al.^32^ which considered the relationship between genotype and functional ERG measures, supports a potential role for altered signalling in cone‐driven OFF pathways in myopia development. As such, measuring retinal function with the ERG could be a useful indicator of future myopia progression. The objective nature of electrophysiology makes it an appealing method for testing retinal function, especially in conditions such as myopia that often develop and progress during childhood. However, this advantage is counterbalanced by the need for invasive recording electrodes, highly trained operators and large and cumbersome equipment. As a result, the widespread adoption of ERG measures beyond research or specialist clinical settings has been limited.

In relation to the first of these disadvantages, an alternative non‐invasive option is the use of skin electrodes. Although skin electrodes record smaller amplitudes compared with the more invasive corneal‐contacting electrodes,^33^, ^34^, ^35^ they offer several advantages. They are easier and quicker to apply, less influenced by blinking and lid twitches^35^ and are better tolerated by participants. Regarding operator skill and cumbersome equipment challenges, a user‐friendly, hand‐held device, the RET*eval®* (LKC Technologies Inc., lkc.com), is now available. This device can obtain fully ISCEV‐compliant ff‐ERGs and has demonstrated high levels of diagnostic agreement with standard ERG systems.^36^, ^37^ The RET*eval®* has been shown to be effective in detecting changes in retinal function associated with various conditions including diabetic retinopathy,^38^, ^39^, ^40^ cone rod dystrophy,^41^ retinopathy of prematurity^42^ and birdshot chorioretinopathy.^43^

Using the RET*eval®* device in combination with skin electrodes addresses several barriers that have previously hindered the widespread adoption of ERGs. However, no study has yet explored whether this approach can effectively detect the previously identified differences in the ERG associated with non‐pathological myopia. Therefore, the objective of this study was to investigate whether this portable device, capable of recording non‐invasive ERG measures when using skin electrodes, can detect differences in retinal function in participants with and without non‐pathological myopia. The study also investigated whether there is a relationship between axial length and ERG measures of retinal function.

## METHODOLOGY

### Participants

**FIGURE 1:**
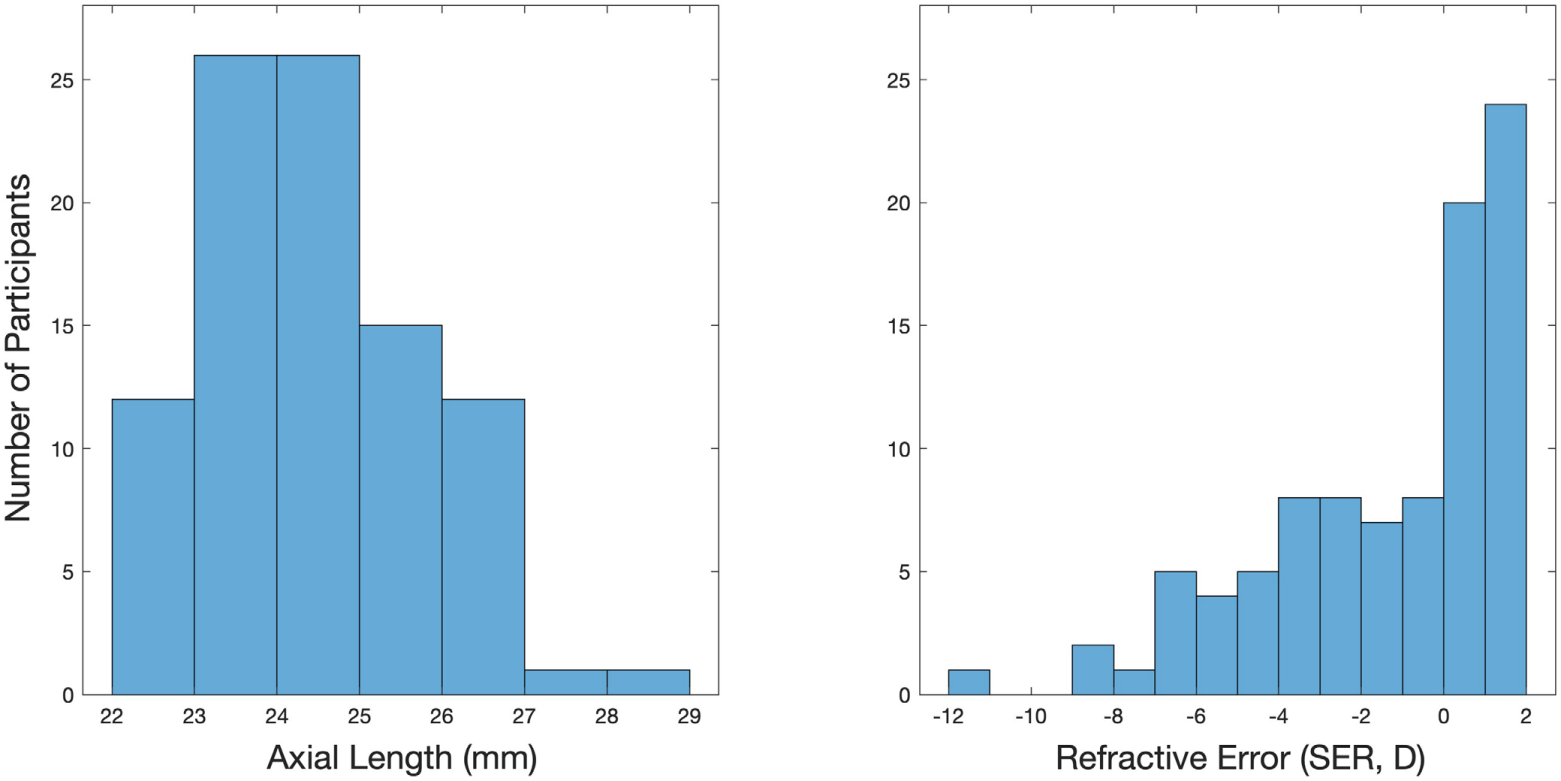
Histograms showing the distribution of axial length and spherical equivalent refractive error (SER) in the study cohort.

Data were obtained from 93 participants, with ages ranging from 18 to 59 years (median 24 years). Participants were separated into two groups, based on their mean spherical equivalent refractive error (SER), measured following the instillation of tropicamide hydrochloride (1.0%). The average of three measurements was captured using a binocular, open‐field autorefractor (Shin Nippon NVision‐K 5001, rexxam.co.jp). Subjects with refractive error ≤ −0.50 DS were classified as ‘myopes’.^6^ The myopic group consisted of 46 participants with SER ranging from −0.50 to −11.25 DS (median −3.75 DS). The non‐myopic control group consisted of 47 participants with SER ranging from +2.00 to −0.25 DS (median +1.00 DS). Axial lengths for each participant were obtained using an IOLMaster 500 (Carl‐Zeiss Meditec, zeiss.com). Three measurements were obtained, and the average was used for analysis. The distribution of axial lengths and refractive errors in the study cohort are displayed in Figure 1.

**FIGURE 2:**
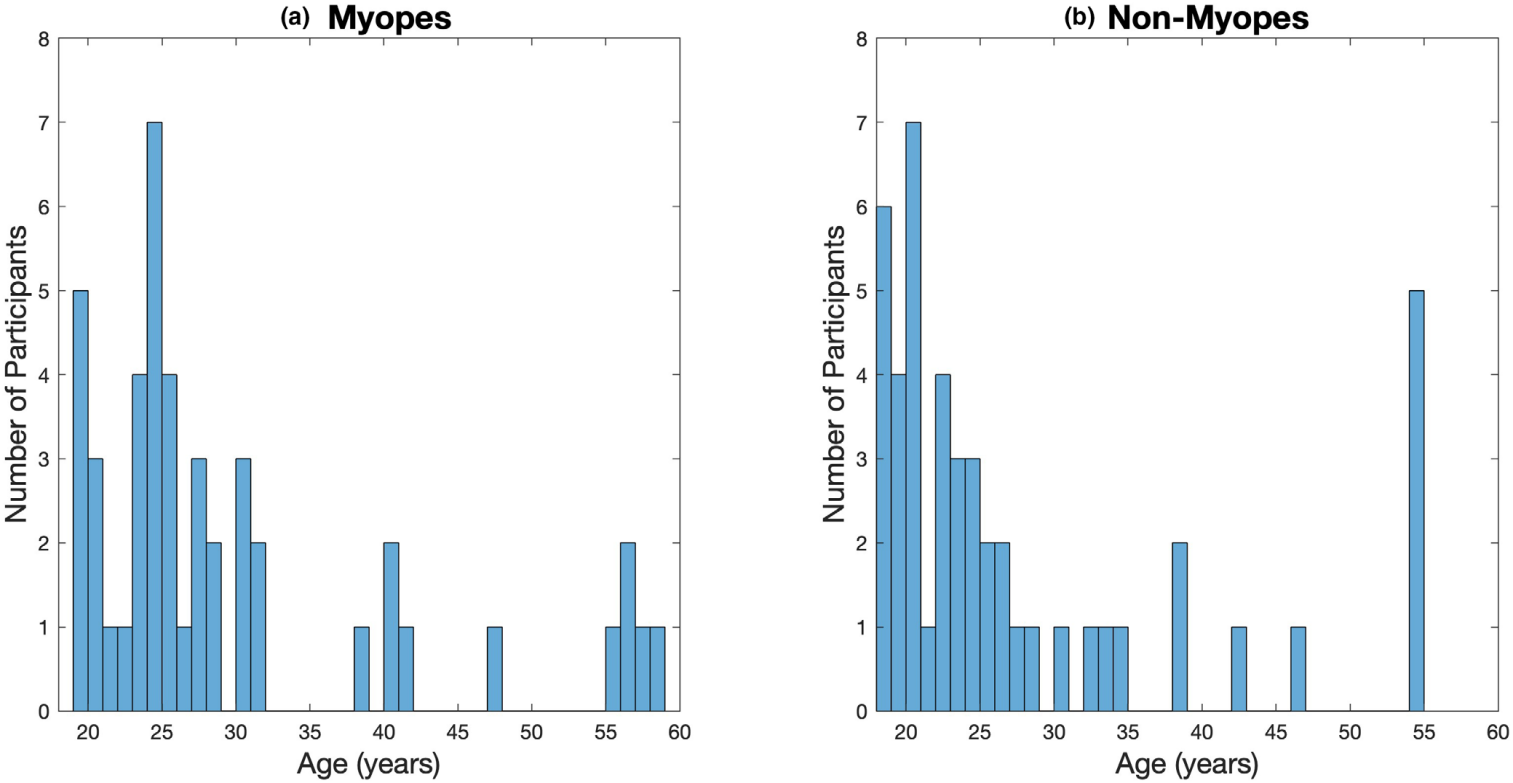
Histograms showing the distribution of age in the myopic and non‐myopic groups.

Participant age was similar in both groups (myopes mean age 29.6 years [range 19–59 years], controls mean age 27.6 years [range 18–55 years], see Figure 2), and the proportion of males and females was similar in both groups (myopic group 33% males, control group 43% males). The characteristics of each group are displayed in Table 1.

**TABLE 1:** Characteristics of the myopic and control groups.

	*n*	Median age (years)	Median SER (DS)	Median astigmatism (DC)	Median AL (mm)	Median average *K* (mm)
Controls	47	23.00 [20.00, 31.50]	+1.00 [+0.50, +1.25]	−0.25 [−0.06, −0.50]	23.59 [23.03, 23.95]	7.79 [7.68, 7.99]
Myopes	46	25.00 [23.00, 31.00]	−3.75 [−2.00, −5.50]	−0.75 [−0.50, −1.25]	25.38 [24.65, 26.53]	7.78 [7.61, 7.87]

*Note*: Summary values are presented as median [IQR].

Abbreviations: AL, axial Length; DC, dioptres cylinder; DS, dioptres sphere; IQR, inter‐quartile range; K, corneal curvature; SER, spherical equivalent refractive error.

The University of Ulster Biomedical Sciences Research Ethics Filter Committee granted ethical approval for this study. The research adhered to the tenets of the Declaration of Helsinki. Informed, written consent was obtained from all participants prior to commencing the study.

Given that no previous study has examined alterations in the ERG amplitudes or implicit time in myopia using the RET*eval*® coupled with skin electrodes, no formal power calculation was undertaken prior to commencing this work. Despite this, the sample was selected to reflect, and in many cases exceed, that included in previous work performed using desktop‐based ERG systems with other forms of electrodes (e.g., Dawson, Trick, and Litzkow [DTL]).

### Screening process

All participants had best‐corrected distance visual acuity of 0.00 logMAR (Snellen equivalent 6/6) or better in both eyes and astigmatism <3.00 DC in the test eye. Intraocular pressure was ≤21 mmHg (as measured with Goldman applanation tonometry or the I‐Care tonometer, icare‐world.com), and no participants had visual field defects when tested using the 24‐2 Swedish Interactive Thresholding Algorithm (SITA) Standard threshold test (Humphrey Visual Field Analyser, Carl Zeiss Meditec, zeiss.com). Anterior and posterior ocular examination was normal, with no abnormalities observed with either macular or peripapillary retinal nerve fibre layer (RNFL) scans captured using a Spectralis optical coherence tomographer (OCT) (Heidelberg Engineering Gmbh., heidelbergengineering.co.uk), nor evident on central and peripheral retinal examination with indirect ophthalmoscopy by a qualified optometrist. No participant had any systemic condition nor was taking any medications that could affect vision. Due to the flashing lights involved with the ERG procedure, individuals with photosensitive epilepsy were excluded from participating in the study.

### Electroretinogram measures

Only one eye (the more myopic eye for the myopia group, selected at random for the control group) from each participant was selected for the experimental measures. The pupil in the test eye was dilated with tropicamide hydrochloride 1.0%, and the constant candela setting was used on the RET*eval*® device (this being denoted by ‘cd’ in test protocols). The protocol selected was the ‘ISCEV 6 Step Dark First cd’, which performs dark‐adapted (DA) tests first, followed by light‐adapted (LA) tests, with each procedure conforming to ISCEV standards. The order of tests was: DA 0.01, DA 3.0, DA 10.0, LA 3.0, LA 3.0 Flicker. The ‘6th step’ refers to the extraction of the oscillatory potentials (OP) from the DA 3.0 ERG.

Prior to commencing, participants were dark adapted for a minimum of 20 minutes in line with McCulloch et al.^44^ At the end of this initial dark adaption period, the skin beneath the test eye was cleaned with NuPrep® gel (weaverandcompany.com), to enhance signal conductivity and aid electrode adherence to the skin, and a Sensor Strip skin electrode (lkc.com) placed 2 mm below the lower eyelid, aligning the inner edge with the middle of the pupil. The skin preparation and electrode application were conducted under dim red light, with an additional dark adaptation period of 5 min provided afterwards, as recommended by the ISCEV standards.^44^ The Sensor Strip contains the active, reference and ground electrodes, and electrical potentials are DC‐amplified and digitised at a sampling rate of 2 kHz.

Throughout the testing procedure, the participant was asked to fixate on the red LED light centred within the Ganzfeld dome. In line with manufacturer guidance the non‐test eye was occluded with the participant's hand to aid stable fixation. The three dark‐adapted tests were completed first, with each test repeated twice before moving on to the next step. Once all dark‐adapted tests were completed, the room lights were switched on and the participant was light adapted for a minimum of 10 minutes, as recommended by ISCEV standards.^44^ The two light‐adapted tests were then completed, with both tests being repeated twice. The average of the two repeats was automatically calculated by the device, and this average was used in subsequent analysis to reduce the effect of measurement noise.

**FIGURE 3:**
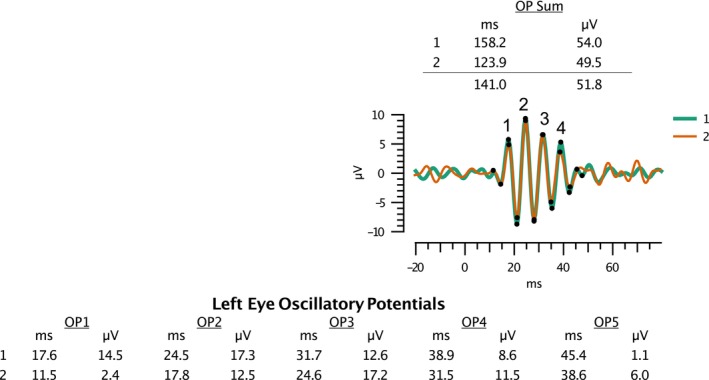
Examples of two repeated dark‐adapted (DA) 3.0 oscillatory potential waveforms for one participant. Note the large differences in reported implicit times/amplitudes between the two waveforms, despite the waveforms overlapping in the diagram. This is because the first cursor (black dot) has been correctly positioned on OP‐1, the first major peak, for waveform one (bold green line), but incorrectly positioned before OP‐1 for waveform two (fine orange line). If the waveform two results are shifted ‘one OP to the left’, then the agreement with waveform one is improved. For clarity, the four main OP wavelets have been labelled. OP, oscillatory potentials.

The amplitude (μV) and implicit time (ms) of the various components of the waveforms were automatically measured and displayed by the RET*eval*® system. The amplitude for the a‐wave was measured from the pre‐stimulus baseline to the a‐wave trough. The b‐wave amplitude was measured from the a‐wave trough to the b‐wave peak. The implicit time of both waves was measured from the time of the flash.^44^ The peaks and troughs of the OP wavelets were also automatically identified by the RET*eval*® device. The implicit time (time to peak) and amplitude (peak to following trough) were then calculated by the RET*eval*® system for each OP wavelet. The automatic peak identification was manually verified by the first author (VS). In some waveforms (*n* = 50/186), the automated identification of the first OP peak was prior to the first major peak, as demonstrated in Figure 3, which shows the two repeated OP measures for an individual participant. Despite the two waveforms overlapping and appearing repeatable in this example, the automatically calculated amplitudes and implicit times were found to be different between the repeat waveforms. In such circumstances (i.e., for any waveform where the first OP was found to be mislabelled before the first peak) the OP‐2 waveform results were taken to be the true OP‐1 results, with the other waveform results also being interpreted in the same manner (i.e., OP‐3 becomes the true OP‐2, etc.). When such adjustments were made, the agreement with the first waveform was improved. This adjustment resulted in the amplitude and implicit time for OP‐5 being unavailable; however, this final wavelet was often unclear and/or not recorded in some participants. The lack of clarity or omission of OP‐5 significantly affected the OP‐Summary implicit time, as it has the longest implicit timing of all wavelet components. To ensure the summary results were comparable across all subjects, OP‐5 results were removed for all participants, and the summary OP values were recalculated for OP‐1 to OP‐4 only. This is in line with ISCEV standards, which state ‘there are usually three main positive peaks often followed by a fourth smaller one’,^44^ and previous ERG studies which have considered OP components 1–4 only.^45^, ^46^, ^47^, ^48^, ^49^, ^50^

### Statistical analysis

For all waveform components, the average of the two repeated tests was used in the analysis.

The absolute value of the amplitude was used (i.e., the negative a‐wave amplitude value was converted into a positive value). Statistical analysis was carried out using MATLAB (2019a, mathworks.com), R (Version 2023.06.1+524, r‐project.org) and SPSS (version 25.0.0.1, ibm.com). An alpha of 0.05 was considered statistically significant, with Holm Bonferroni correction applied to adjust for multiple comparisons. For clarity, both the raw and corrected *p*‐values (post‐hoc Holm‐Bonferroni correction) are reported in the results tables, with only the corrected *p*‐values shown in the figures.

The Shapiro–Wilk test was used to assess the normality of the data. As the data did not follow a normal distribution, the median amplitude and implicit value for both the myopic and control groups were obtained and compared using the Mann–Whitney *U*‐test.

The relationship between axial length and ERG output parameters was examined using Kendall's tau correlation and Passing‐Bablok regression (transformation method). This regression technique was chosen as it is: (i) suitable for non‐parametric data, (ii) permits error in both the x and y variables and (iii) has been shown to be less influenced by the presence of outliers and yield more precise estimates of slope and intercept compared with ordinary least squares or Deming regression.^51^, ^52^ Two assumptions of the Passing‐Bablok analysis are that the relationship between the x and y variables is linear and that there is a significant positive correlation between the two variables. These were tested using a cumulative sum (cusum) and Kendall's tau correlation tests, respectively.^53^ The relationship between axial length, rather than refractive error, and ERG output was investigated given that axial length is not affected by optical components (e.g., corneal curvature) and thus has been deemed a more appropriate parameter to use for accurate interpretation of the ERG response in myopic cohorts.^15^

## RESULTS

### Comparison of ERG amplitudes between myopes and non‐myopes

The amplitude data (μV) for each waveform component is displayed in Table 2. While the median amplitude for the myopic group was slightly greater than the controls for all components with the exception of OP‐4, differences in amplitude between the myopic and control groups failed to reach statistical significance for any ERG component post Holm‐Bonferroni correction.

**TABLE 2:** Electroretinogram (ERG) amplitudes (μV) for each step of the six‐step International Society for Clinical Electrophysiology of Vision (ISCEV) protocol.

	Myope	Non‐myope	*p*‐Value	Corrected *p*‐value
**DA 0.01**				
B‐wave	21.5 [14.3–30.8]	20.4 [14.6–29.1]	0.70	>0.99
**DA 3.0**				
A‐wave	27.7 [16.9–36.7]	26.1 [18.7–30.8]	0.32	>0.99
B‐wave	44.5 [30.2–63.5]	40.7 [30.6–48.7]	0.26	>0.99
**DA 10.0**				
A‐wave	37.0 [25.5–50.6]	33.4 [26.0–41.2]	0.25	>0.99
B‐wave	48.8 [34.5–65.2]	45.6 [37.4–50.9]	0.32	>0.99
**DA OP**				
Summary	42.4 [30.1–52.9]	40.2 [31.2–57.1]	0.97	>0.99
1	12.9 [10.0–15.8]	12.7 [10.1–16.3]	0.76	>0.99
2	15.1 [10.1–17.7]	13.3 [10.2–20.7]	0.89	>0.99
3	10.2 [6.7–14.1]	9.7 [6.6–12.1]	0.63	>0.99
4	5.1 [3.0–7.4]	5.3 [2.8–7.1]	0.98	>0.99
**LA 3.0**				
A‐wave	6.3 [4.2–7.8]	4.7 [3.7–6.0]	0.03*	0.10
B‐wave	30.9 [24.8–38.9]	25.6 [17.5–35.3]	0.06	0.12
**LA flicker**	25.3 [20.8–35.0]	24.3 [19.4–36.5]	0.70	0.70

*Note*: Results displayed as median [IQR] for myopes and non‐myopic controls. The *p*‐value was calculated using the Mann–Whitney *U*‐test with Holm‐Bonferroni correction then applied (where a *p*‐value is highlighted with a ‘*’ that denotes statistical significance is reached).

Abbreviations: DA, dark adapted; IQR, inter‐quartile range; LA, light adapted; OP, oscillatory potentials.

### Comparison of ERG implicit times between myopes and non‐myopes

**FIGURE 4:**
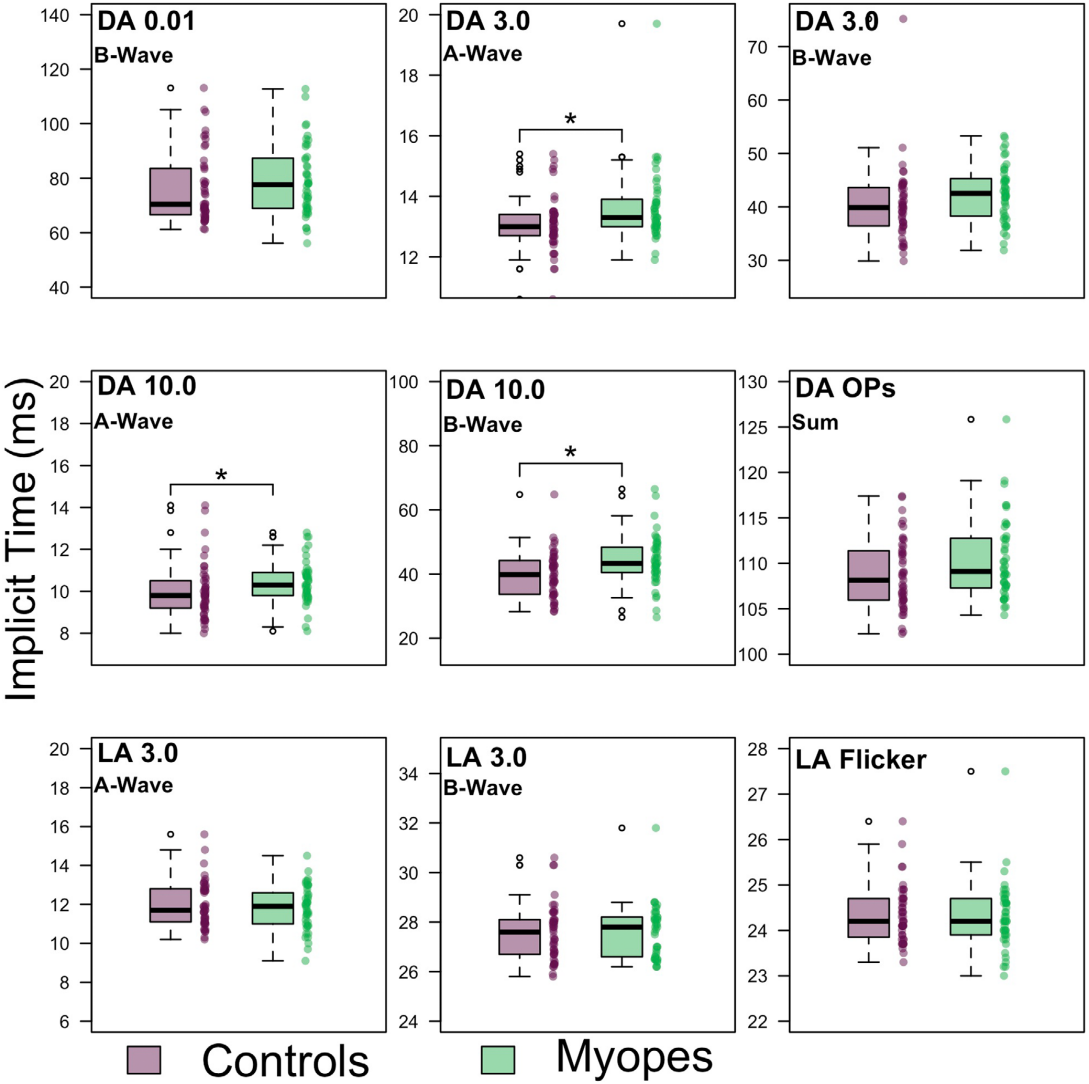
Electroretinogram (ERG) implicit times for each step of the six‐step International Society for Clinical Electrophysiology of Vision (ISCEV) protocol in myopes and controls. Individual data points are shown next to boxplots. The ‘*’ denotes significance for Mann–Whitney *U*‐test at *p* < 0.05 with Holm‐Bonferroni correction applied. DA, dark adapted; LA, light adapted; OP, oscillatory potentials.

The median implicit time (ms) for all dark‐adapted components was longer in the myopic group compared with the control group, and the difference reached statistical significance (post Holm‐Bonferroni correction) for the DA 3.0 A‐wave, DA 10.0 A‐wave, DA 10.0 B‐wave, DA OP‐1 and the DA OP‐2. There were no significant differences between the median light‐adapted implicit times for myopes and controls. The implicit time data are displayed in Table 3 and illustrated in Figure 4.

**TABLE 3:** Electroretinogram (ERG) implicit times (ms) for each step of the six‐step International Society for Clinical Electrophysiology of Vision (ISCEV) protocol.

	Myope	Non‐myope	*p*‐Value	Corrected *p*‐value
**DA 0.01**				
B‐wave	77.6 [68.9–87.3]	70.4 [66.5–83.8]	0.15	0.15
**DA 3.0**				
A‐wave	13.3 [13.0–13.9]	13.0 [12.7–13.4]	0.007*	0.04*
B‐wave	42.6 [38.3–45.3]	39.9 [36.4–43.8]	0.02*	0.05
**DA 10.0**				
A‐wave	10.3 [9.8–10.9]	9.8 [9.2–10.5]	0.009*	0.04*
B‐wave	43.3 [40.4–48.6]	39.8 [33.7–44.2]	0.005*	0.03*
**OP**				
Summary	109.1 [107.3–112.8]	108.2 [105.9–111.4]	0.07	0.14
1	17.0 [16.7–17.5]	16.7 [16.4–17.0]	0.004*	0.02*
2	23.9 [23.4–24.5]	23.3 [23.0–23.9]	0.01*	0.03*
3	30.5 [30.0–31.7]	30.0 [29.5–31.3]	0.07	0.14
4	38.0 [36.9–39.6]	38.0 [36.5–39.0]	0.39	0.39
**LA 3.0**				
A‐wave	11.9 [11.0–12.7]	11.7 [11.1–12.8]	0.81	>0.99
B‐wave	27.8 [26.6–28.2]	27.6 [26.7–28.1]	0.74	>0.99
**LA flicker**				
	24.2 [23.9–24.7]	24.2 [23.8–24.7]	0.96	>0.99

*Note*: Results are displayed as median (IQR) for myopes and non‐myopic controls. The *p*‐value was calculated using the Mann–Whitney *U*‐test and has been adjusted for multiple comparisons using Holm‐Bonferroni correction (where a *p*‐value is highlighted with a ‘*’ this reached statistical significance).

Abbreviations: DA, dark adapted; IQR, inter‐quartile range; LA, light adapted; OP, oscillatory potentials.

### Relationship between axial length and functional ERG output

**FIGURE 5:**
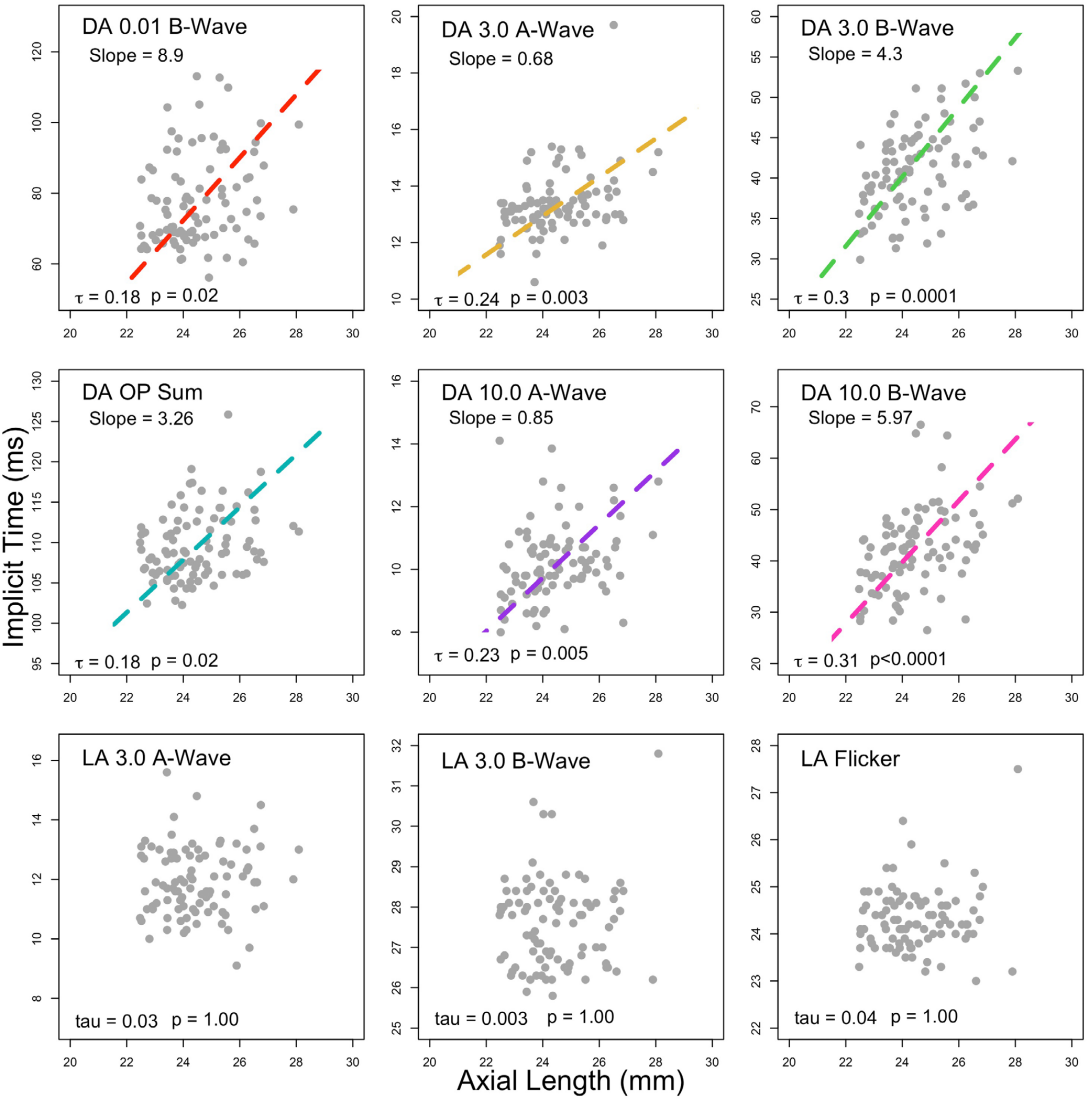
The relationship between axial length and implicit time. A Passing Bablok regression line is plotted (coloured dashed lines) when requirements are met, including a significant positive coefficient with Kendall's tau correlation. *p*‐Values stated in the figure are post Holm‐Bonferroni correction. DA, dark adapted; LA, light adapted; OP, oscillatory potentials.

For all dark‐adapted tests, there was a significant, moderate positive correlation between axial length and implicit time, with longer eyes having significantly longer implicit times. The light‐adapted implicit times did not show any relationship with axial length. The results of the Kendall's tau correlations between axial length and implicit time are displayed in Table 4 and graphically in Figure 5.

**TABLE 4:** Correlation analysis for axial length and electroretinogram (ERG) amplitude and implicit time data.

	Axial length and amplitude			Axial length and implicit time		
	Tau	*p*‐Value	Corrected *p*‐value	Tau	*p*‐Value	Corrected *p*‐value
**DA 0.01**						
B‐wave	0.08	0.27	0.54	0.18	0.009*	0.02*
**DA 3.0**						
A‐wave	0.11	0.11	0.39	0.24	0.0009*	0.003*
B‐wave	0.12	0.10	0.39	0.30	<0.0001*	0.0001*
**DA 10.0**						
A‐wave	0.14	0.04*	0.27	0.23	0.002*	0.005*
B‐wave	0.14	0.05	0.27	0.31	<0.0001*	<0.0001*
**OP**						
Sum	−0.02	0.77	0.77	0.18	0.01*	0.02*
1	0.02	0.82	>0.99	0.22	0.002*	0.007*
2	−0.01	0.87	>0.99	0.19	0.008*	0.02*
3	0.02	0.78	>0.99	0.19	0.008*	0.02*
4	−0.05	0.45	>0.99	0.15	0.04*	0.04*
**LA 3.0**						
A‐wave	0.18	0.01*	0.04*	0.03	0.67	>0.99
B‐wave	0.15	0.04*	0.08	0.003	0.96	>0.99
**LA flicker**	0.10	0.17	0.17	0.04	0.60	>0.99

*Note*: The results are stated as the Kendall's tau correlation coefficient and the associated *p*‐value with Holm‐Bonferroni correction applied (where a ‘*’ denotes statistical significance).

Abbreviations: DA, dark adapted; LA, light adapted; OP, oscillatory potentials.

**FIGURE 6:**
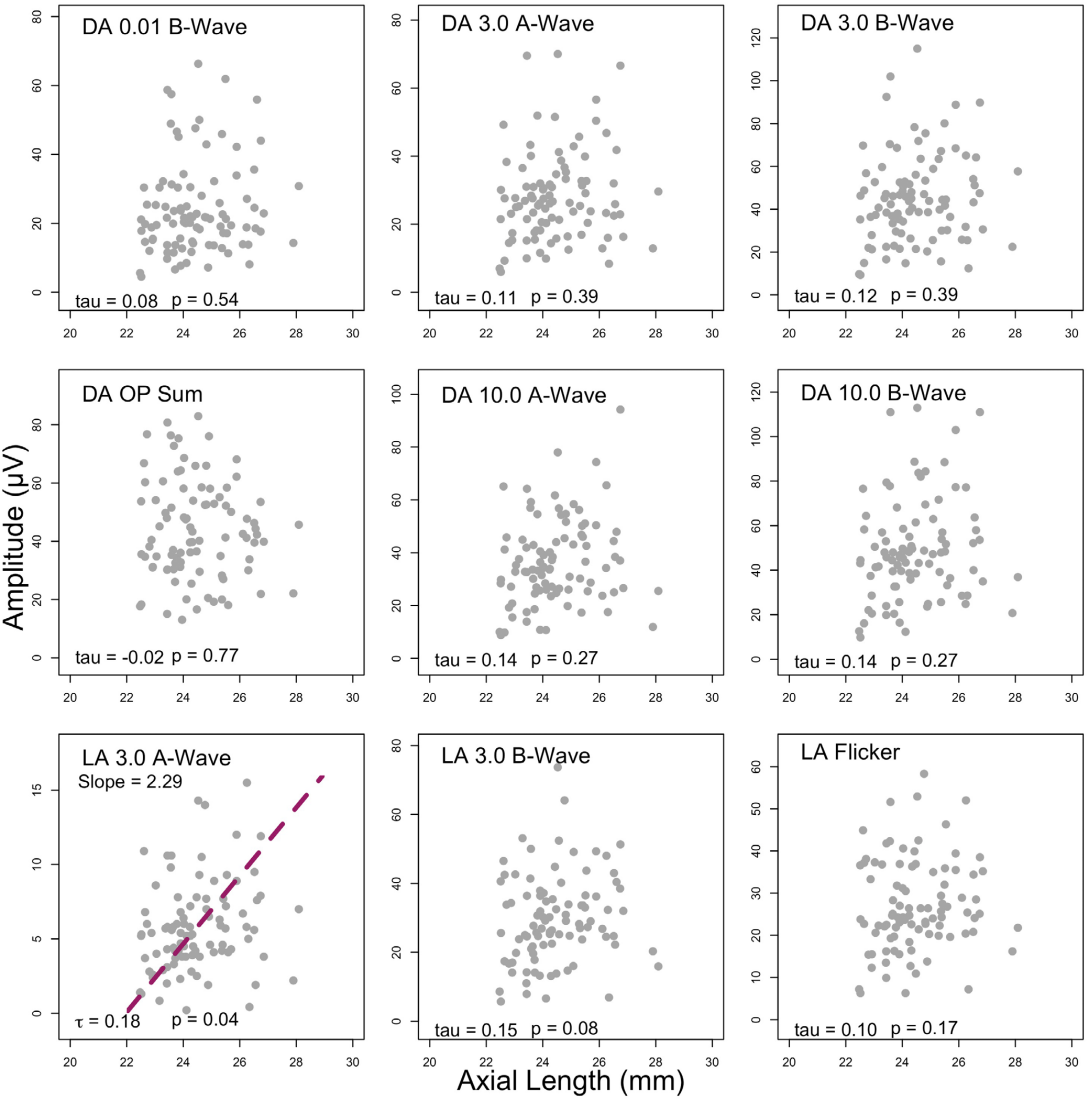
The relationship between axial length and amplitude. A Passing Bablok regression line is plotted (coloured dashed line) when requirements are met, including a significant positive coefficient with Kendall's tau correlation. *p*‐Values stated in the figure are post Holm‐Bonferroni correction. DA, dark adapted; LA, light adapted; OP, oscillatory potentials.

For all ERG components other than the dark‐adapted OPs, there was a weak positive correlation between axial length and amplitude. The correlations did not, however, reach statistical significance for any component other than the LA A‐Wave (*p* = 0.04). The Kendall's tau values for the OP components indicated an extremely weak relationship between these variables and axial length, with these also failing to reach statistical significance. The results of the Kendall's tau correlations between axial length and amplitude are displayed in Table 4 and graphically in Figure 6.

## DISCUSSION

Understanding the impact of myopia on retinal function offers the potential to identify functional biomarkers, which could help identify those at risk of myopic changes^30^, ^31^ or be useful in understanding the effects and safety of novel myopic control strategies.^10^, ^11^, ^12^ Previous reports using conventional ERG setups with either corneal contact‐lens (e.g., Burian‐Allen^15^, ^18^, ^27^ or Jet^28^) or conjunctival (e.g., DTL,^16^, ^17^, ^20^, ^22^, ^23^, ^24^, ^25^, ^26^, ^30^, ^31^, ^32^ Gold foil^19^ or H‐K [Hawlina‐Konec] loop^29^) electrodes have demonstrated reductions in ERG amplitude^13^, ^14^, ^15^, ^16^, ^17^, ^18^, ^19^, ^20^, ^21^, ^22^, ^23^ and delays to the ERG response (implicit time) in myopia.^16^, ^20^, ^21^, ^23^, ^24^, ^25^, ^26^, ^27^, ^28^, ^29^ This study investigated whether such changes to the ERG in myopia could be detected by a hand‐held device (RET*eval®*) and non‐invasive skin electrodes. With this equipment, subtle delays in the ERG timing (implicit time) were observed, with significant positive correlations between axial length and dark‐adapted implicit. However, no significant differences were found in light‐adapted implicit times nor response amplitudes under any adaptation conditions between the myopic and non‐myopic groups, nor any associations between these measures and axial length (with the exception of a weak positive relationship between axial length and the LA 3.0 A‐wave amplitude).

### Amplitude of response

These findings of delayed implicit times, but no changes to amplitude, in myopia align with two previous studies using the mf‐ERG^25^ and PERG.^24^ However, the vast majority of previous reports supported a significantly lower ERG amplitude in myopia,^13^, ^14^, ^15^, ^16^, ^17^, ^18^, ^19^, ^20^, ^21^, ^22^, ^23^ and therefore one must consider why the RET*eval*® and skin electrode setup used in this study did not detect amplitude differences in myopia.

One possible explanation is that the amplitude measurements were variable, with the result that any alterations in myopia (i.e., the myopia ‘signal’) became lost in the measurement variability (noise). To build support for this explanation, one should consider both the ‘signal’ and the ‘noise’ components of the study measurements. In terms of the myopia ‘signal’, this is likely to be small in this myopic cohort given they did not have pathological myopia, and subjects were included with a wide range of myopic refractive errors, including those with only mild to moderate levels of myopia (Figure 1). Participants with such myopic refractive errors were chosen for this study given our interest in the potential use of the RET*eval*® and skin electrode setup to detect or predict myopic onset and progression. However, the relatively small number of high myopes (*n* = 9 with refractive error < −6.00 D) limits any sub‐analysis to determine whether the expected reduction in ERG amplitudes would be evident for higher levels of myopia. Indeed, when considering the statistically significant delays in dark‐adapted implicit time observed in the myopic group, while statistically significant, the delays were only small (range 0.3–3.5 ms compared with the control group, depending upon the waveform component considered), suggesting only subtle differences in retinal function in this myopic cohort. The correlations observed between axial length and implicit time do indicate that those with higher levels of myopia exhibited greater differences in retinal function, and thus the myopia ‘signal’ would likely be higher if only (or a greater sample of) those with high myopia were considered. Future work could focus on high myopia, to ascertain whether ERG amplitude is reduced in this population.

In terms of the measurement noise, amplitude measurements are known to be more variable and less reproducible than implicit time measures.^54^ For example, Chen et al.^25^ suggested their findings of altered implicit time, but not amplitude, could be due to the greater degree of inter‐subject variability in their amplitude data. The use of a skin electrode in the present study will further increase the potential for noisy amplitude measures as there are more opportunities for physiological and extraneous interference.^33^ Indeed, when comparing a skin electrode to a DTL electrode, Mortlock et al.^55^ found that while implicit time variability was similar and low for both electrode types, inter‐individual variation for amplitudes was significantly greater when skin electrodes were used. The recorded response amplitude can also be adversely affected by the positioning of the skin electrode, whereas implicit times appear less influenced by electrode positioning.^56^ While care was taken to be concise and consistent with electrode positioning in the present study, even small discrepancies in position between subjects could exacerbate the already variable amplitude responses achieved with skin electrodes. To our knowledge, this is the first study investigating the ERG in myopia using a skin electrode, and so the points made above regarding skin electrode variability and positioning are likely to have contributed to the small myopia ‘signal’ being lost in the noise of the measurement.

However, it is notable that these amplitude results not only generally failed to reach statistical significance but also exhibited trends that were in the opposite direction to that expected. Here, median amplitudes were slightly higher in the myopic group relative to controls, and most components exhibited a weak, but positive, correlation with axial length. This result has parallels to an earlier study by Sannita et al.^57^ who suggested the use of a skin electrode led to an unexpected finding of a positive correlation between b‐wave amplitude and age, which was contradictory to the negative correlations reported by other studies using corneal electrodes. The authors suggested that changes to ocular components or to the skin surrounding the orbit with age could affect the electrical resistance, and therefore the signal picked up by the skin electrode. It is possible that a similar hypothesis may be applied to the present study, whereby orbital anatomy differences in myopic observers relative to controls affected the electrical resistance and thus the signal picked up by the skin electrode. Potential anatomical factors in myopia that could affect the amplitude of the signal picked up by the skin electrode include less orbital fat/tissue and a thinner anterior sclera. To our knowledge, no previous study has looked specifically at orbital fat in myopia, but former work indicates that despite the eye growing in myopia, the size of the orbit is largely the same,^58^, ^59^, ^60^ which could leave less space in the myopic orbit for fat or soft tissue if no proptosis occurs. In terms of scleral thickness, research has primarily concentrated on posterior scleral thinning in myopia.^61^, ^62^ Dhakal et al.^63^ measured anterior scleral thickness and found the inferior sclera to be significantly thinner with increasing magnitude of myopia, which is interesting in the context of this study given the inferior positioning of the skin electrode.

It is also worth considering that the present study was conducted cross‐sectionally on adult myopes, and different results may present for a child/adolescent cohort, particularly given this is the age range where actively progressing myopia is most likely to occur. A longitudinal study by Luu et al.^30^ found that mfERG amplitude was significantly reduced at baseline only in those children (aged 9–11 years at baseline) whose myopia progressed rapidly (>0.50 D/year) over the subsequent two‐year period. In addition, a recent study involving electrophysiology and genotyping^32^ reported differences in cone‐driven a‐wave amplitude in those with specific alleles associated with a genetically greater risk of myopia progression. As such, the findings reported for adults in this study may not reflect what would occur in those with actively progressing myopia and/or those who have this specific genetic predisposition to myopia development/progression. The ability to stratify participants by their genotypic risk for myopia development and/or progression would be valuable in future work to determine how visual function measures are altered in myopic individuals and at what stage in the myopia development they occur.

### Timing of response

The RET*eval*® and skin electrode setup allowed the detection of small, but significant, delays in implicit time for some dark‐adapted components in myopia. In addition, axial length was significantly correlated with the implicit time of all dark‐adapted components; a larger axial length was associated with a longer implicit time. These correlations were mild to moderate (Kendall's tau ranged from 0.15 to 0.31). Central axial length was used as a surrogate measure of myopic structural change, rather than posterior segment shape, which may be a more appropriate structural correlate for the ff‐ERG, which represents the average functional response of the retina.^64^ It would be valuable for future research to explore associations between posterior segment shape (e.g., as can be measured using magnetic resonance imaging (MRI) or though peripheral ocular length measurements) and ff‐ERG timing in myopia.

To explore whether dark‐adapted implicit times have potential as a functional biomarker for predicting future myopia onset or progression, additional work is required on a child cohort including a longitudinal study on a group of progressive myopes. Furthermore, using genotyping to stratify future cohorts as to their genetic risk of myopic progression^32^ would be interesting. While the objective nature of the ERG, combined with the portability of the RET*eval*® device and non‐invasive nature of the skin electrodes, are attractive attributes, the period of dark adaptation required may render dark adapted implicit times unrealistic as a clinical test for myopia management and monitoring, particularly in children.

When considering light‐adapted waveforms, no significant differences were found between refractive groups, nor any relation between axial length and implicit times. While this lack of association suggests these more easily achievable measures will not be helpful as functional biomarkers for myopia management, the contrast between scotopic and photopic retinal function in myopia is interesting and aligns with a recent study by Wan et al.^65^ They found that purely rod‐driven OPs were affected in myopia, but that the photopic, cone‐driven responses were unaffected. A trend for greater effects on scotopic compared with photopic waveforms in myopia has also been reported in analyses of the ERG amplitude.^16^, ^17^, ^18^

In terms of explanations as to why there would be greater changes to the scotopic compared with the photopic response in myopia, one possibility is that the process of dark adaptation is altered in myopia and that this subsequently affects the dark‐adapted ERG tests. Indeed, previous work has shown a correlation between elevated dark‐adapted rod thresholds and ERG response deficits in other ocular conditions.^66^ There are very few studies considering dark adaptation in non‐pathological myopia, and these reported only small effects on the dark adaptation process in high myopia.^67^, ^68^ These studies used subjective psychophysical tests, and it is possible that larger changes to dark adaptation may become apparent using objective methodology, such as the ERG.

Another possibility is that there is an underlying dysfunction of the rod pathway in myopia.^65^ The rod pathway interacts both structurally and functionally with the dopaminergic system,^69^ with dopamine playing a key role in modulating the rod pathway.^70^ For example, dopamine modulates the electrical coupling and spatial integration of the A‐II amacrine cell,^71^ a key interneuron in the scotopic pathway that most, if not all, scotopic signals from rods must pass through.^72^ Given there is evidence of reduced retinal dopamine levels contributing to myopia development and progression,^73^, ^74^, ^75^ it is conceivable that changes to the dopamine system in myopia could contribute to the alterations in rod‐mediated visual function seen here and in other investigations.

### The influence of age on results

Previous studies have found that age can affect the ERG response, with generally a reduced amplitude^76^, ^77^, ^78^, ^79^, ^80^, ^81^, ^82^, ^83^, ^84^ and longer implicit time^78^, ^80^, ^81^, ^82^, ^83^, ^84^ occurring with older age. However, for participants between 20 and 60 years of age, Park et al.^27^ found that age had no significant effect on the mf‐ERG responses. In addition, the reductions in amplitude and delay in timing of the ff‐ERG reported by Parvaresh et al.^81^ for a cohort aged 1–80 years were only present for the oldest, i.e., 70–80 year age group. The age range of our participants was thus limited to 18–59 years, chosen to maintain a maximal representative sample from ‘healthy adult’ participants. All potential participants underwent a thorough ocular examination prior to data collection to ensure any age‐related pathologies were excluded. Age was also similar across the myopic and non‐myopic control groups (myopes: mean age 29.6 years [range 19–59 years], controls: mean age 27.6 years [range 18–55 years], see Figure 2).

To rule out the possibility that the inclusion of participants aged 18–59 years could have affected the conclusions drawn from this study, two additional post‐hoc age‐related analyses were conducted. First, the comparative analysis of the myopic and non‐myopic ERG outputs was repeated with a restricted sample of only those aged 18–40 years. Second, the effect of age (as a potential confounding factor) on the observed relationships between axial length and ERG output was considered using multiple linear regression (MLR). The results of these analyses are described in detail in the S1 Data.

In brief, when the sample was restricted to those aged 18–40 years, the same trends apply compared with the whole group analysis. The myopic group had longer dark‐adapted implicit times compared to non‐myopic controls, significant (*p* < 0.05) for DA 3.0 A‐wave, DA 3.0 B‐wave, DA 10.0 A‐wave, DA 10.0 B‐Wave, OP‐1, OP‐2 and OP‐3. Following Holm‐Bonferroni correction, statistically significant differences remained for the DA 3.0 A‐Wave (*p* = 0.02), OP‐1 (*p* = 0.04) and OP‐3 (*p* = 0.03) ERG components. However, other DA implicit time comparisons failed to reach statistical significance post Holm‐Bonferroni correction (DA 10.0 B‐wave: *p* = 0.06, DA 3.0 A‐Wave: *p* = 0.07, DA 10.0 B‐wave: *p* = 0.07, OP‐Sum: *p* = 0.07, OP‐3: *p* = 0.07), this likely reflecting the reduced sample size of the restricted 18–40‐year‐old cohort (14 participants [15%] removed). As found in the full cohort, there were no significant differences in light‐adapted implicit times nor response amplitudes between myopes and non‐myopes in the restricted 18–40 year cohort.

In terms of whether age (potential confounding independent variable) is influencing the relationships observed between axial length (target independent variable) and implicit time (outcome dependent variable), MLR was used. Details of the MLR model and results are provided in the S1 Data. The results show that the moderate, positive correlations between axial length and dark‐adapted implicit time were still evident when age was accounted for. The absolute value of the coefficient between axial length and dark‐adapted implicit time was, however, slightly lower after adjustment for age; prior to accounting for age the coefficient for axial length and dark‐adapted implicit time ranged from 0.18 to 0.45 (mean 0.32 ± 0.08), whereas after accounting for age the coefficient ranged from 0.12 to 0.44 (mean 0.25 ± 0.10). Following the adjustment for age, the relationships observed between axial length and dark‐adapted implicit time were all still statistically significant pre‐Holm Bonferroni correction, with the majority also remaining significant post correction. However, post‐Holm‐Bonferroni correction, the relationship of four components (DA 0.01‐B, DA OP‐Sum, DA OP‐1, DA OP‐2) with axial length failed to reach statistical significance (*p* = 0.08 [both DA 0.01‐B and DA OP‐Sum] or *p* = 0.06 [both DA OP‐1 and DA OP‐2]). This likely reflects that a larger sample is needed to consider fully the effect of both age and axial length on these specific ERG components.

In summary, while age does also appear to affect implicit times in the current cohort, the main trends reported between axial length and implicit time still stand when age is considered as a factor in the analysis.

## CONCLUSIONS

The RET*eval®* hand‐held ERG device, used in conjunction with a skin electrode, did not detect the reduction in amplitude previously reported in myopic eyes using traditional ERG setups and contact electrodes. This is likely because the small myopic ‘signal’ was masked in the noise of measurement resulting from high inter‐subject variability and/or anatomical confounders associated with the use of a skin electrode. However, subtle differences in dark‐adapted implicit timing were evident using this portable and non‐invasive setup. Further research is required to explore whether such measures are relevant and informative in myopia management.

## AUTHOR CONTRIBUTIONS

**Victoria Stapley:** Conceptualization (equal); data curation (lead); formal analysis (lead); investigation (lead); methodology (equal); project administration (lead); resources (supporting); visualization (lead); writing – original draft (lead). **Roger S. Anderson:** Conceptualization (equal); formal analysis (supporting); methodology (supporting); resources (equal); supervision (equal); writing – original draft (supporting). **Kathryn Saunders:** Conceptualization (supporting); formal analysis (supporting); methodology (supporting); resources (equal); supervision (equal); writing – original draft (supporting). **Pádraig J. Mulholland:** Conceptualization (equal); formal analysis (supporting); investigation (supporting); methodology (equal); project administration (supporting); resources (equal); supervision (lead); visualization (supporting); writing – original draft (supporting).

## FUNDING INFORMATION

This work was supported by a PhD studentship from the Department for the Economy, Northern Ireland (VS) and by the National Institute for Health and Care Research Biomedical Research Centre at Moorfields Eye Hospital NHS Foundation Trust and UCL Institute of Ophthalmology (RSA, PJM). LKC Technologies provided a loan of the RET*eval®* for the duration of this study, and travel support to present the findings of this study at the EU‐RETINA conference (VS). The views expressed are those of the authors and not necessarily those of the National Health Service, the National Institute for Health and Care Research or the Department of Health.

## CONFLICT OF INTEREST STATEMENT

LKC Technologies provided a loan of the RETeval device and travel support to Victoria Stapley (VS). This is already included here. Otherwise no author had a conflict of interest.

## Supporting information


Data S1:
