# Supplementary material for: Measuring differences in the ERG in myopia using the RETeval device with skin electrodes
Source: Ophthalmic Physiol Opt. 2025 Feb 13;45(3):666–80. doi: 10.1111/opo.13460 (PMC11976514; doi:10.1111/opo.13460)
Supplement: Supplementary file 1 — Data S1: [file OPO-45-666-s001.docx]

**Supplementary Materials for:**

**Measuring differences in the ERG in myopia using the RETeval device and Skin Electrodes**

**Running Title:** ERG in myopia with RETeval device and skin electrodes

**Authors Names:**

Victoria Stapley,^1^ Roger S. Anderson,^1,2^ Kathryn Saunders,^1^ Pádraig J. Mulholland^1, 2^

^1^ Centre for Optometry and Vision Sciences, Biomedical Sciences Research Institute, Ulster University, Coleraine, UK.

^2^ National Institute for Health and Care Research (NIHR) Biomedical Research Centre, at Moorfields Eye Hospital NHS Foundation Trust and UCL Institute of Ophthalmology, London, UK.

**Repeat analysis with restricted 18-to-40 years cohort**

The reduced 18-to-40 years cohort consisted of 79 participants; 39 myopes (SER ranging from -0.50DS to –11.25DS, median -3.75DS), and 40 non-myopes (SER ranging from +2.00DS to -0.25DS, median +0.75DS). The median amplitude and implicit time for each waveform component was calculated and reported in Table S1, with comparisons between the myopic and non-myopic groups (Mann-Whitney UTest) also being reported. A p-value of <0.05 was considered statistically significant and denoted in table S1 with an asterisk. P-values were then adjusted for multiple comparisons using Holm-Bonferroni correction.

Full interpretation of results is included in main manuscript.

| **Waveform** | **Myope Median [IQR]** | **Non-Myope Median [IQR]** | **p-value** | **Corrected p-value** |
| --- | --- | --- | --- | --- |
| **Implicit Time** |  |  |  |  |
| DA 0.01 B-Wave | 74.7 [68.4 to 83.5] | 69.4 [65.9 to 78.8] | 0.13 | 0.13 |
| DA 3.0 A-Wave | 13.3 [13.0 to 13.8] | 12.9 [12.6 to 13.4] | 0.003* | 0.02* |
| DA 3.0 B-wave | 42.1 [38.4 to 45.1] | 39.9 [36.4 to 43.4] | 0.02* | 0.07 |
| DA 10.0 A-Wave | 10.3 [9.8, 10.7] | 9.8 [9.0 to 10.5] | 0.02* | 0.07 |
| DA 10.0 B-Wave | 43.2 [40.1 to 47.6] | 38.9 [33.7 to 43.8] | 0.01* | 0.06 |
| DA OP-Sum | 108.8 [107 to 112.5] | 107.1 [105.7 to 110.9] | 0.03* | 0.07 |
|  | | | | |
| DA OP-1 | 16.9 [16.6 to 17.2] | 16.6 [16.3 to 16.9] | 0.01* | 0.04* |
| DA OP-2 | 23.8 [23.3 to 24.2] | 23.3 [23.0 to 23.8] | 0.007* | 0.03* |
| DA OP-3 | 30.3 [29.9 to 31.4] | 29.8 [29.5 to 31.0] | 0.04* | 0.07 |
| DA OP-4 | 38.0 [36.9 to 39.5] | 37.5 [36.4 to 38.8] | 0.17 | 0.17 |
|  | | | | |
| LA 3.0 A-Wave | 11.9 [10.9 to 12.5] | 11.6 [11.0 to 12.7] | 0.75 | 1.00 |
| LA 3.0 B-Wave | 27.3 [26.5 to 28.1] | 27.3 [26.6 to 28.1] | 0.97 | 1.00 |
| LA Flicker | 24.2 [23.9 to 24.6] | 24.1 [23.8 to 24.7] | 0.84 | 1.00 |
|  | | | | |
| **Amplitude** |  |  |  |  |
| DA 0.01 B-Wave | 20.0 [14.0 to 29.7] | 20.8 [14.4 to 27.9] | 0.98 | 1.00 |
| DA 3.0 A-Wave | 25.9 [16.5 to 34.4] | 25.8 [19.3 to 30.9] | 0.81 | 1.00 |
| DA 3.0 B-wave | 43.9 [30.3 to 57.3] | 39.5 [31.5 to 49.8] | 0.52 | 1.00 |
| DA 10.0 A-Wave | 34.1 [25.4 to 46.8] | 33.9 [26.5 to 41.6] | 0.73 | 1.00 |
| DA 10.0 B-Wave | 45.6 [33.2 to 61.5] | 44.9 [35.0 to 51.8] | 0.74 | 1.00 |
| DA OP-Sum | 42.3 [31.0 to 52.8] | 44.2 [31.2 to 60.4] | 0.61 | 1.00 |
|  |  |  |  |  |
| DA OP-1 | 13.3 [9.8 to 16.8] | 12.9 [10.1 to 17.1] | 0.94 | 1.00 |
| DA OP-2 | 14.9 [10.1 to 17.4] | 13.3 [10.2 to 21.5] | 0.75 | 1.00 |
| DA OP-3 | 10.1 [6.7 to 12.5] | 9.7 [6.6 to 14.0] | 0.91 | 1.00 |
| DA OP-4 | 5.1 [3.0 to 7.3] | 5.2 [2.8 to 7.2] | 0.88 | 1.00 |
|  |  |  |  |  |
| LA 3.0 A-Wave | 5.9 [3.8 to 7.8] | 4.5 [3.7 to 6.0] | 0.13 | 0.38 |
| LA 3.0 B-Wave | 31.5 [24.8 to 38.5] | 25.7 [17.6 to 35.9] | 0.16 | 0.38 |
| LA Flicker | 25.7 [20.7 to 35.1] | 24.5 [21.3 to 36.7] | 0.98 | 0.98 |

**Table S1**: ERG amplitudes and implicit times for each step of the 6-step ISCEV protocol, for a restricted cohort aged 18-to-40 years. Results displayed as median [IQR] for myopes and non-myopic controls. The p-value was calculated using the Mann-Whitney U-test with Holm-Bonferroni correction applied. Where a p-value is highlighted with a ‘*’ this reached statistical significance.

**Relationship between Age and ERG outputs**

To consider whether age was significantly affecting the ERG output in our cohort, Kendall’s tau correlation was performed between age and the amplitude and implicit time of each waveform, with the results reported in Table S2.

For amplitude, correlation coefficient ranged from weak positive (0.11 for DA 10 B-wave) to weak negative (-0.12 for DA OP-1). No correlation was significant even before Holm-Bonferroni correction.

For implicit time, there was a weak to moderate positive relationship with age for all components (tau range from 0.11 to 0.27), other than DA 3.0 B-wave and LA Flicker where there was no relationship (tau of 0.04 for both). Post Holm-Bonferroni correction, the correlations remained statistically significant for DA 0.01 B-wave, DA 3.0 A-wave and all OP components (all p<0.05).

|  | **Correlation Age and Amplitude** | | | **Correlation Age and Implicit Time** | | |
| --- | --- | --- | --- | --- | --- | --- |
| **ERG Component** | Tau | p-value | Corrected p-value | Tau | p-value | Corrected p-value |
| DA 0.01 B-Wave | 0.01 | 0.87 | 1.00 | 0.18 | 0.01* | 0.04* |
| DA 3.0 A-Wave | 0.03 | 0.68 | 1.00 | 0.20 | 0.006* | 0.03* |
| DA 3.0 B-wave | 0.02 | 0.80 | 1.00 | 0.04 | 0.59 | 0.60 |
| DA 10.0 A-Wave | 0.06 | 0.42 | 1.00 | 0.15 | 0.05 | 0.15 |
| DA 10.0 B-Wave | 0.11 | 0.14 | 1.00 | 0.11 | 0.14 | 0.28 |
| DA OP-Sum | -0.05 | 0.45 | 1.00 | 0.27 | 0.0002* | 0.001* |
|  | | | | | | |
| DA OP-1 | -0.12 | 0.11 | 0.44 | 0.19 | 0.01* | 0.01* |
| DA OP-2 | -0.02 | 0.76 | 1.00 | 0.23 | 0.002* | 0.008* |
| DA OP-3 | -0.02 | 0.76 | 1.00 | 0.22 | 0.003* | 0.008* |
| DA OP-4 | -0.03 | 0.72 | 1.00 | 0.22 | 0.002* | 0.008* |
|  | | | | | | |
| LA 3.0 A-Wave | 0.05 | 0.53 | 1.00 | 0.16 | 0.04* | 0.11 |
| LA 3.0 B-Wave | 0.01 | 0.85 | 1.00 | 0.12 | 0.11 | 0.22 |
| LA Flicker | -0.08 | 0.28 | 1.00 | 0.04 | 0.63 | 0.63 |

**Table S2**: Outcomes of Kendall’s tau correlation examining the relationship between (a) participant age and ERG amplitudes, and (b) participant age and ERG implicit times. Both uncorrected and correct p-values (following Holm-Bonferroni correction) are listed, with a ‘*’ highlighting those values for which a statistically significant correlation was observed.

We then sought to investigate whether age could be cofounding the relationships between axial length (independent target variable of interest) and ERG implicit time (dependent outcome variable) reported in the main manuscript. The dependent outcome variable of ERG amplitude was not considered given the lack of associations observed between ERG amplitude and both age and axial length.

**Does age affect the relationships observed between axial length and implicit time?**

To consider whether age could be affecting our observed relationships between axial length and implicit time, we performed a multiple linear regression analysis (SPSS, Version 29.0.2.0). ‘Implicit time’ was entered as the dependent variable. ‘Axial length’ and ‘Age’ were entered as independent variables.

Prior to reporting the results of this analysis, the assumptions for multiple linear regression (MLR) were checked for each waveform component. If any data point had a standardised residual with an absolute value of >3.0, it was considered a potential outlier ^1^. The decision to remove the data point from the analysis was based on whether this was also skewing the normality of the residuals. The normality of the residuals was assessed by plotting a Q-Q plot and using a Shapiro-Wilk test. Data was assumed to not follow a normal distribution if the Shapiro-Wilk p-value was <0.05. If a data point had a standardised residual >3.0 and the normality of residuals was not met (Shapiro Wilk p<0.05), then the data was deemed an outlier, removed, and the MLR re-run. The normality of the residuals was then re-checked. If the residuals were now deemed normally distributed (Shapiro Wilk p>0.05) then results of the MLR were reported. If the residuals were still deemed not normally distributed (Shapiro Wilk p<0.05) the results of the MLR were not reported. Details of the decision process for each waveform component are outlined in Table S3. In all cases, removal of the outlier did not affect the overall trend, nor significance of, the reported relationship between axial length and implicit time (Table S4).

| **Implicit Time** | **Standardised residual above 3?** | **Normality of residuals** | **Decision** |
| --- | --- | --- | --- |
| DA 0.01 B-Wave | No | P=0.05 (borderline) | Run MLR on full dataset |
| DA 3.0 A-Wave | Yes, 1 participant (value 5.4) | Pre p<0.001, Post p=0.93 | Run MLR without 1 participant data |
| DA 3.0 B-wave | Yes, 1 participant (value 5.8) | Pre p<0.001, Post p=0.11 | Run MLR without 1 participant data |
| DA 10.0 A-Wave | Yes, 1 participant (value 5.8) | Pre p=0.001, Post p=0.14 | Run MLR without1 participant data |
| DA 10.0 B-Wave | Yes, 1 participant (value 3.2) | Pre p=0.21 | Run MLR on full dataset |
| DA OP-Sum | Yes, 1 participant (value 5.8) | Pre p=0.001, Post p=0.12 | Run MLR without 1 participant data |
| DA OP-1 | Yes, 1 participant (value 3.5) | Pre p=0.09 | Run MLR on full dataset |
| DA OP-2 | Yes, 2 participants (value 3.7 and 4.1) | Pre p<0.001, Post p=0.67 | Run MLR without 2 participant data |
| DA OP-3 | Yes, 1 participant (value 5.1) | Pre p<0.001, Post p=0.005 | Do not report MLR |
| DA OP-4 | Yes, 1 participant (value 3.4) | Pre p=0.04, Post p=0.39 | Run MLR without 1 participant data |
| LA 3.0 A-Wave | Yes, 1 participant (value 3.4) | Pre p=0.25 | Run MLR on full dataset |
| LA 3.0 B-Wave | Yes, 1 participant (value 3.9) | Pre p<0.0001, Post p=0.01 | Do not report MLR |
| LA Flicker | Yes, 2 participant (value 4.4 and 3.1) | Pre p<0.001, Post p=0.71 | Run MLR without 2 participant data |

**Table S3**: Decision process for removal of outliers and running multiple linear regression (MLR) for each wave-form component. Data point removed if standardised residual >3 and if p<0.05 when testing normality of residuals with Shapiro Wilk (‘pre’ = pre removal of outlier(s)). MLR re-run without data point and normality of residuals rechecked with Shapiro Wilk (‘post’ = post removal of outlier(s)). MLR only reported if test for normality now p>0.05.

|  | **Relationship between axial length and implicit time** | | | |
| --- | --- | --- | --- | --- |
| **Implicit Time** | 1. **MLR on full dataset** | | 1. **MLR on dataset with outlier(s) removed** | |
|  | **Coefficient** | **P<0.05** | **Coefficient** | **P<0.05** |
| DA 0.01 B-Wave | 0.20 | Y | No outlier identified | |
| DA 3.0 A-Wave | 0.29 | Y | 0.27 | Y |
| DA 3.0 B-wave | 0.33 | Y | 0.44 | Y |
| DA 10.0 A-Wave | 0.22 | Y | 0.30 | Y |
| DA 10.0 B-Wave | 0.34 | Y | 0.35 | Y |
| DA OP-Sum | 0.22 | Y | 0.19 | Y |
| DA OP-1 | 0.22 | Y | 0.22 | Y |
| DA OP-2 | 0.21 | Y | 0.21 | Y |
| DA OP-4 | 0.15 | N | 0.12 | N |
| LA 3.0 A-Wave | 0.002 | N | No outlier identified | |
| LA Flicker | 0.12 | N | -0.05 | N |

**Table S4**: Standardised coefficient and associated p-value for the relationship between axial length and implicit time reported from a MLR ran on the dataset pre (A) and post (B) removal of any outlier(s) as outlined in Table S3.

The outcomes of MLR analyses performed (including standardised coefficient, and associated p-values for the relationship between axial length and implicit time) are reported in table S5-A. This can be interpreted as the relationship between axial length (target independent variable) and implicit time (outcome dependent variable) when the independent variable ‘age’ is held constant. Or in other words, represents the association between axial length and implicit time, adjusting/accounting for age.

We also compared the association (standardised coefficient and p-value) between axial length and implicit time found in the MLR (i.e. when age is accounted for) from that found from a simple linear regression (SLR) between these two factors (i.e. when age is not accounted for). These results are also reported in table S5.

From table S5-A we can see that the moderative, positive correlations between axial length and dark-adapted implicit time are still evident when age is accounted for. Comparing table S5-A (after accounting for age) and S5-B (prior to accounting for age), we can see that the absolute value of the coefficient between axial length and dark-adapted implicit time is slightly lower after the adjustment for age. Further interpretation of results included in main manuscript.

We should also note here that the correlation coefficients and p-values reported from the SLR of axial length and implicit time in table S5-B are not exactly the same as those reported for the same variables in table 4 of the main manuscript. This is not unexpected given the different forms of correlation analysis carried out. However, importantly, the trends remain the same. The Kendall’s tau correlation coefficients reported in the main manuscript are all slightly lower (i.e. more conservative) than the Pearson’s rho correlation coefficients reported here by the SLR. Reported p-values if deemed statistically significant were all at the same level (p<0.05), other than OP-4 which was marked statistically significant (p=0.04) for Kendall’s tau correlation in the main manuscript, and not statistically significant (p=0.09) for the SLR reported here.

|  | **A. Regression of axial length and implicit time from MLR (i.e. accounting for age)** | | | **B. Regression of axial length and implicit time from SLR (i.e. not accounting for age)** | | |
| --- | --- | --- | --- | --- | --- | --- |
|  | **Coefficient** | **P value** | **Corrected p-value** | **Coefficient** | **P value** | **Corrected p-value** |
| **DA 0.01 B-Wave** | 0.20 | 0.04* | 0.08 | 0.27 | 0.01* | 0.02* |
| **DA 3.0 A-Wave** | 0.27 | 0.003* | 0.009* | 0.34 | 0.001* | 0.003* |
| **DA 3.0 B-wave** | 0.44 | 0.00002* | 0.0001* | 0.45 | 0.000006* | 0.00004* |
| **DA 10.0 A-Wave** | 0.30 | 0.002* | 0.008* | 0.36 | 0.0006* | 0.002* |
| **DA 10.0 B-Wave** | 0.34 | 0.0004* | 0.002* | 0.40 | 0.00009* | 0.0005* |
| **DA OP-Sum** | 0.19 | 0.04* | 0.08 | 0.27 | 0.01* | 0.02* |
|  | | | | | | |
| **DA OP-1** | 0.22 | 0.02* | 0.06 | 0.29 | 0.005* | 0.02* |
| **DA OP-2** | 0.21 | 0.02* | 0.06 | 0.30 | 0.005* | 0.02* |
| **DA OP-3** | Violated assumptions of MLR | | | | | |
| **DA OP-4** | 0.12 | 0.24 | 0.24 | 0.18 | 0.09 | 0.09 |
|  | | | | | | |
| **LA 3.0 A-Wave** | 0.002 | 0.98 | 1.00 | 0.05 | 0.51 | 1.00 |
| **LA 3.0 B-Wave** | Violated assumptions of MLR | | | | | |
| **LA Flicker** | -0.05 | 0.67 | 1.00 | -0.01 | 0.91 | 1.00 |

**Table S5**: (A) The association between axial length and implicit time when age is accounted for using MLR. (B) The association between axial length and implicit time using SLR, when the effect of age is not accounted for. The standardised coefficients and p-values (uncorrected and corrected following Holm-Bonferroni correction) are listed. A ‘*’ highlights a significance level of p<0.05.

**Additional References**

1. Wang Y, Lin L, Thompson CG, Chu H. A penalization approach to random-effects meta-analysis. Stat Med. 2022;41(3):500-16. Epub 20211118.
